# Endotoxin Molecule Lipopolysaccharide-Induced Zebrafish Inflammation Model: A Novel Screening Method for Anti-Inflammatory Drugs

**DOI:** 10.3390/molecules19022390

**Published:** 2014-02-21

**Authors:** Li-Ling Yang, Guo-Quan Wang, Li-Mei Yang, Zhi-Bing Huang, Wen-Qing Zhang, Lin-Zhong Yu

**Affiliations:** 1 School of Traditional Chinese Medicine, Southern Medical University, Guangzhou 510515, China; E-Mails: yll138@126.com (L.-L.Y.); wgq585@163.com (G.-Q.W.); 2 School of Laboratory Medicine, Guangdong Medical College, Dongguan 523808, China; E-Mail: yanglm2@163.com; 3 The Key Laboratory of Zebrafish Modeling and Drug Screening for Human Diseases Institute, Southern Medical University, Guangzhou 510515, China; E-Mail: huangzb20@163.com

**Keywords:** zebrafish, LPS, macrophages, neutrophils, screening, qRT-PCR, mortality

## Abstract

Lipopolysaccharide (LPS), an endotoxin molecule, has been used to induce inflammatory responses. In this study, LPS was used to establish an *in vivo* inflammation model in zebrafish for drug screening. We present an experimental method that conveniently and rapidly assesses the anti-inflammatory properties of drugs. The yolks of 3-day post-fertilization (dpf) larvae were injected with 0.5 mg/mL LPS to induce fatal inflammation. After LPS stimulation, macrophages were tracked by NR and SB staining and neutrophil migration was observed using the MPO:GFP line. Larval mortality was used as the primary end-point. Expression levels of key cytokines involved in the inflammatory response including IL-1β, IL-6, and TNF-α, were measured using quantitative reverse transcription polymerase chain reaction (RT-PCR). Macrophages and neutrophils were both recruited to the LPS-injected site during the inflammatory response. Mortality was increased by LPS in a dose-dependent manner within 48 h. Analyses of IL-1β, IL-6, and TNF-α expression levels revealed the upregulation of the inflammatory response in the LPS-injected larvae. Further, the anti-inflammatory activity of chlorogenic acid (CA) was evaluated in this zebrafish model to screen for anti-inflammatory drugs. A preliminary result showed that CA revealed a similar effect as the corticosteroid dexamethasone (DEX), which was used as a positive control, by inhibiting macrophage and neutrophil recruitment to the LPS site and improving survival. Our results suggest that this zebrafish screening model could be applied to study inflammation-mediated diseases. Moreover, the Traditional Chinese Medicine CA displays potential anti-inflammatory activity.

## 1. Introduction

Lipopolysaccharide (LPS) is an endotoxin molecule and the major constituent of the outer membrane of all Gram-negative bacteria [1]. LPS elicits directly or indirectly multiple pathophysiological processes *in vivo*, such as metabolic changes, fever, multiple organ dysfunction syndrome (MODS), endotoxic shock, and death in extreme cases [2,3]. These effects are associated with the stimulation of LPS to receptors which mediate the signaling cascades and then lead to the activation of neutrophils and macrophages and the release of inflammatory cytokines such as IL-6, IL-8, and TNF-α [4].

Although there have been improvements in the mortality of systemic inflammatory response syndrome (SIRS) owing to the supportive care and the use of antibiotics, accumulating evidence has also demonstrated that some Traditional Chinese medicines (TCMs) or their components could protect mice against endotoxins [5,6]. Studies showed that the natural compounds cloud attenuate LPS-induced inflammatory responses in MODS, such as chlorogenic acid (CA), genipin [7,8]. These compounds have been shown to be beneficial for the treatment of inflammatory response in inflammation-related diseases.

Many experimental methods and animal models have been established for the determination of the numerous LPS-mediated biological effects on a host [9,10,11]. These models fulfill many of the accepted requirements for developing anti- inflammatory strategies through genetics, immunology, and pharmacology. At present, mammals are most suitable to model endotoxicity. However, different animal species vary in sensitivity to LPS, such as rabbits and horses are the most sensitive while rats and mice are the least [9,10]. Furthermore, conspecific animals exhibit dramatic differences in lethal doses by LPS and a wide variation in responses under different experimental conditions [12,13]. Therefore, an appropriate experimental model system should be chosen independently based on the experimental aims of each study.

Zebrafish, with their unique advantages in biology, genomics, and genetics, and their high conservation of signal transduction pathways linked to disease, have been used as a model for human disorders [14,15]. Notably, the pathological features in terms of inflammation of zebrafish were similar to those of humans. In recent years, zebrafish have been established as an ideal model for the pathophysiology of human inflammatory-related diseases and high-throughput *in vivo* screening. The transparency of zebrafish embryos and larvae considerably simplifies the analysis of the hematological system. In addition, many transgenic zebrafish types have been established through labeling inflammatory immunocytes, including early myeloid cells [16], neutrophils [17,18] and early macrophages [19]. Moreover, the zebrafish model was more suitable than cells or rodent models for high-throughput drug screening [20]. Additionally, the zebrafish has been considered as an alternative model organism for disease modeling and drug discovery and has further been applied for the reduce, refine, replace (3R) concept [21].

In this study, we established a LPS-induced zebrafish inflammation model through injecting LPS to induce an acute inflammatory response. Furthermore, dexamethasone (DEX) and chlorogenic acid (CA) were used to confirm the reliability of the LPS-induced zebrafish inflammation model by inhibiting inflammatory neutrophil and macrophage recruitment, as well as reducing LPS-induced mortality. This study provided a novel model for the screening of anti-inflammatory agents.

## 2. Results and Discussion

In the present study, we showed that the injection of LPS could induce the inflammatory responses via increasing the expression levels of the inflammatory cytokines including IL-1β, TNF-α, and IL-6. These cytokines resulted in a significant inflammatory damage and death in zebrafish larvae. Furthermore, we were able to evaluate rapidly the anti-inflammatory effects of DEX and CA on neutrophils and macrophages by observing the changes in their migration and number, the quantitative expression of inflammatory cytokines, and mortality rates.

**Figure 1:**
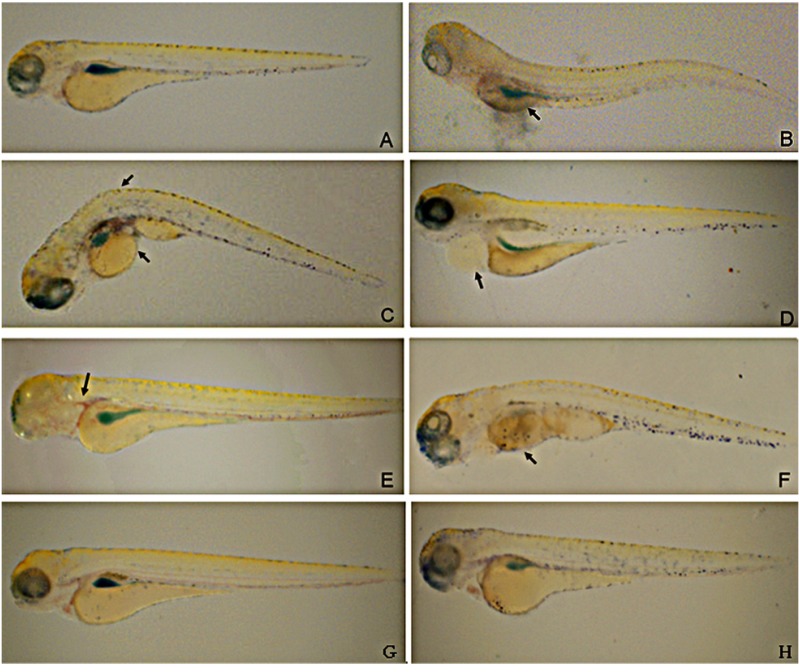
The induction of LPS on inflammation phenotype in zebrafish. The yolks of zebrafish larvaes were injected with 0.5 mg/mL LPS to induce reproducible morphological abnormalities at 24 h. Figure **B**–**F** present the observed morphological abnormalities. (**B**) Necrotic yolk; (**C**) yolk crenulation and cyrtosis; (**D**) swollen pericardium sac; (**E**) hemorrhagic pericardium; (**F**) death. (**A**) PBS injection was used for negative control while (**G**) un-treated zebrafish larvaes served as normal control. Larvae exposed to 0.5 mg/mL LPS by immersion for 24 h (**H**). The representative images have been taken from three individual experiments (*n* = 34).

### 2.1. LPS Causes Abnormalities in Zebrafish Larvae

LPS can stimulate host defense mechanisms and induce fatal inflammation [22]. In order to characterize the zebrafish inflammation response towards LPS, we injected LPS (0.5 mg/mL, 2 nL volume per larva) into the yolk of 3-day post-fertilization (dpf) larvae (*n* = 34). At 24 hpi, we found that 0.5 mg/mL LPS (2 nL volume per larva) caused reproducible morphological abnormalities (Figure 1B,F). Larvae displaying yolk necrosis, cyrtosis, pericardium hemorrhage or dying were considered to be severely affected. The numbers of larvae with abnormalities increased with the extension of post-injection time. The percentages of larvae with abnormalities at 24 h are listed in Table 1. These results indicate that the injection of LPS into the yolk of 3-dpf zebrafish larvae causes the changes in morphology and increases mortality. In contrast, larvae exposed to 0.5 mg/mL LPS by immersion did not show obvious toxic phenotypes nor increased mortality within 24-h post-exposure (hpe; Figure 1H).

**Table 1:** Larval phenotype statistics.

Group	Phenotype ^a^ (%)					
	Normal	Necrotic yolk	Cyrtosis	Swollen pericardial sac	Hemorrhagic pericardium	Death
Negative control	100	/	/	/	/	/
Yolk injection	/	22.0 ± 0.9	30.7 ± 0.2	4.6 ± 0.5	12.6 ± 0.5	34.0 ± 1.5

^a^ Data pooled from three independent experiments with an average n of 34 fish per group.

In order to develop an objective, high-performance, and rapid method to screen anti-inflammatory drugs in the zebrafish, we identified and compared three important factors that influenced the survival: (1) route of injection; (2) timing of LPS injection; and (3) LPS dose.

### 2.2. Comparision of Immersion and Injection for Inflammatory Responses

#### 2.2.1. Inflammatory Macrophage Migration (NR Staining)

**Figure 2:**
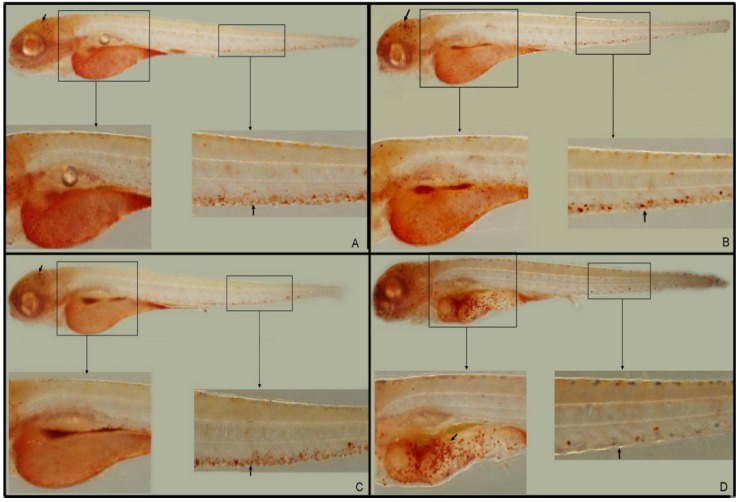
Representative pictures of NR labeling of macrophages at 3.5 dpf. (**A**) Normal untreated controls; (**B**) zebrafish larvae immersed in LPS (0.5 mg/mL); (**C**) PBS-injected negative controls; and (**D**) larvae with LPS (0.5 mg/mL) injected into the yolk (*n* = 34).

The macrophages that arise from the anterior lateral plate mesoderm are capable of phagocytosing cellular debris, apoptotic cell corpses, and microbes. These macrophages can be injected into the blood circulation or into one of the closed body cavities, such as the hindbrain ventricle [23,24]. Macrophages stimulate lymphocytes and other immune cells to respond to pathogens, for instance, via the regulation of TNF-α, IL-1, and IL-6. Macrophage lysosomes fuse to form a single large, red vacuolar aggregate, allowing easy detection of these cells in the larvae by NR staining [25]. Larvae used for NR staining studies were treated at 3-dpf and sampled at 4 hpi or hpe. In 3.5-dpf larvae, macrophages predominantly appeared in the midbrain region and blood islands (PBI; Figure 2A,C arrow). In LPS-immersed larvae, no abnormal red vacuolar aggregates (*i.e.*, macrophages) were observed in non-yolk larval tissues (Figure 2B) when compared with the microinjection of PBS (Figure 2C). However, in larvae microinjected with LPS, large red vacuolar aggregates appeared in the yolk sac, with almost no staining in the PBI (Figure 2D, arrow). Thus, macrophages appear to leave the brain and PBI and migrate to the inflammatory foci in the yolk of LPS-injected larvae. These findings demonstrated that LPS could induce the inflammatory responses in zebrafish larvae and the injection was more suitable for the induction of inflammation responses in zebrafish larvae.

#### 2.2.2. Neutrophil Migration for Inflammatory Responses (SB Staining)

**Figure 3:**
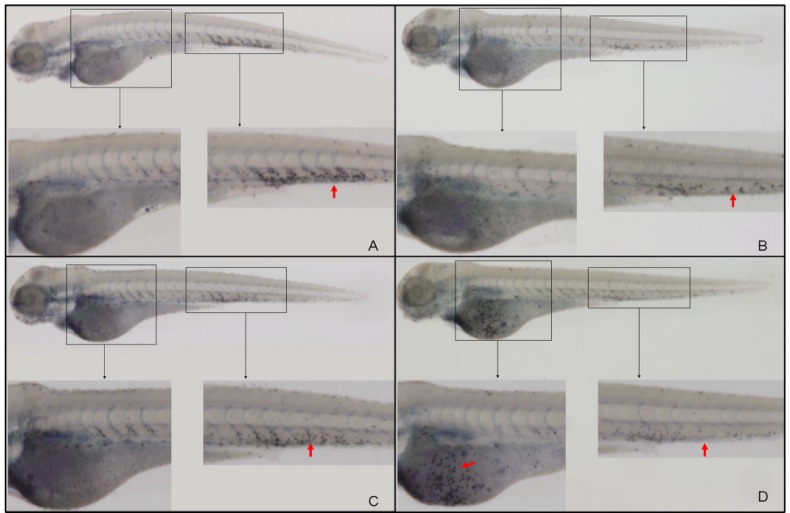
Representative pictures of SB labeling of neutrophils at 3 dpf. (**A**) Normal controls; (**B**) larvae immersed in LPS (0.5 mg/mL); (**C**) PBS-injected negative controls; and (**D**) larvae with LPS (0.5 mg/mL) injected into the yolk (*n* = 34).

Sudan black (SB) and MPO staining of neutrophils have been developed for the evaluation of inflammation responses in mammals [26,27]. In this study, SB staining was performed to observe neutrophil migration during acute inflammation in zebrafish for the first time. 3-dpf larvae were fixed at 6 hpi or 6 hpe. From normal 2–4 dpf larvae, neutrophils were predominantly localized to the area of the PBI, with fewer cells in the head stroma and epidermis (Figure 3A, arrow). After being injected with LPS, large and clear cytolymph lipid droplets (*i.e.*, neutrophils; Figure 3D, arrow) were present in the yolk sac. However, for LPS-immersed larvae, there was a slight increase in staining in the epidermis (Figure 3B). In both injected and immersed larvae, neutrophils in the PBI were reduced compared with the controls (Figure 3C. arrow), with a slightly stronger effect in injected larvae. These results suggest that LPS injection induces the recruitment of neutrophils from the vasculature to the foci. However, the injection manner was more suitable than immersion for the induction of inflammation in zebrafish larvae. The percentages of larvae with leukocytes recruited to the yolk are presented in Table 2.

**Table 2:** Yolk leukocyte recruitment statistics.

Group	Occurrence Rate ^a^ (%)		
	NR Staining		SB Staining
Normal control		0	0
Immersion		0	0
Negative control		5.6 ± 0.3	3.3 ± 0.6
Yolk injection		96.6 ± 0.6	95.5 ± 0.9

^a^ Data pooled from three independent experiments with an average *n* = 34 fish per group.

#### 2.2.3. Tracking the Neutrophil Inflammatory Response using the MPO: GFP Line

**Figure 4:**
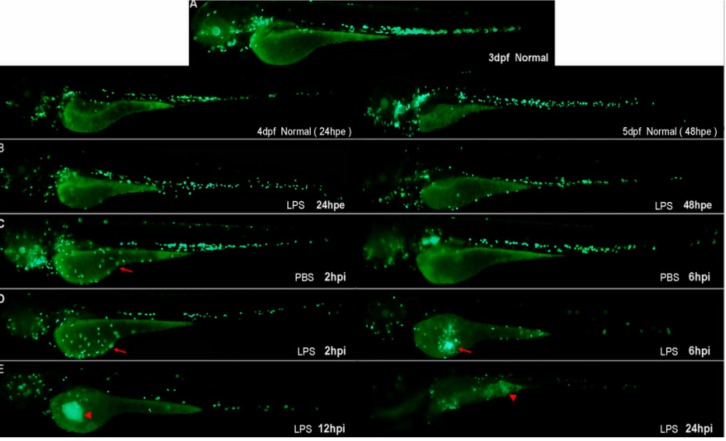
Representative pictures of MPO:GFP lines reveal the LPS-modeled infection process at 3 dpf. (**A**) Normal controls (untreated with any agents or operation); (**B**) larvae immersed in LPS (0.5 mg/mL); (**C**) PBS-injected negative controls; and (**D**) larvae with LPS (0.5 mg/mL) injected into the yolk (*n* = 34).

Neutrophil infiltration is characteristic of acute inflammation. It has been reported that wounding induces the recruitment of neutrophils from the vasculature to the wound site. The majority of neutrophils that enter the wound quickly return to the vasculature, while a minority migrate around the wound site, then return to the vasculature after inflammation regresses [17,28]. Using the MPO:GFP line [17], no abnormal recruitment of neutrophils was observed in LPS-immersed larvae within 48 hpe compared with controls (Figure 4B). A few neutrophils were found at the wound at 2 hpi in the PBS-injected negative controls and LPS-injected larvae (Figure 4C,D arrow). At 6 hpi, neutrophils migrated from the wound site back to the vasculature in PBS-injected larvae (Figure 4C). However, in LPS-injected larvae, neutrophils were robustly retained at the injection site (Figure 4D, arrow). LPS doses below 0.125 mg/mL produced similar results (data not shown). By 12–16 hpi, a fluorescent signal circled the neutrophil recruitment site (Figure 4E, arrowhead). By 18–24 hpi, most of the larvae showed yolk necrosis, including a few deaths (Figure 4E, arrowhead). 

#### 2.2.4. Survival Rates

**Figure 5:**
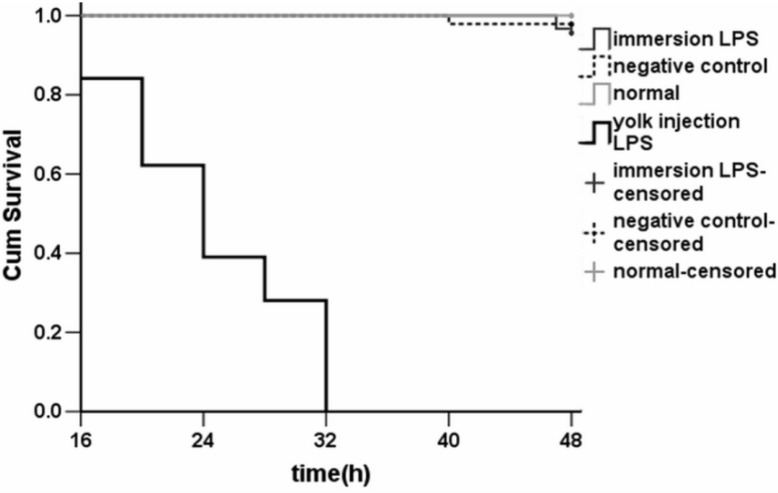
Average cumulative mortality (percent) of 3-dpf zebrafish larvae by different routes of LPS exposure. 3-dpf zebrafish larvae were exposed to 0.5 mg/mL LPS by immersion (*n* = 34, *p* < 0.001) or injection into the yolk (*n* = 34, *p* < 0.001), and monitored for 48 h. Negative controls were injected with PBS. Each curve represents data pooled from three independent experiments.

Larvae from the same mother were randomly divided into experimental and control groups. Then 3-dpf larvae were exposed by immersion or microinjection with 0.5 mg/mL LPS, and then maintained at 28.5 °C. Injection of LPS caused 100% mortality within 32 h, whereas larvae injected with the same volume (2 nL) of PBS survived (Figure 5). Most of the larvae had an obviously toxic phenotype at 24 h, exhibiting necrotic yolks, cyrtosis, malformed tails, swollen pericardial sacs, hemorrhagic pericardiums, or bradycardia. In contrast, larvae immersed in LPS exhibited 100% survival at 45 h without any obvious toxic phenotypes (Figure 5). 

The survival time of LPS-injected larvae was significantly less than that of LPS-immersed larvae (*p* < 0.001, *n* = 34). Consistent with the findings of Bates and Akerlund [1], these results demonstrate that LPS exposure by immersion has little effect on 3-dpf larvae. The usual methods of exposing zebrafish embryos to foreign agents include microinjection or static immersion [29]. In an ideal model system, inflammation would have high mortality and obvious inflammatory phenotypes. While LPS immersion (0.5 mg/mL) causes very low mortality and no obvious inflammation phenotypes, the injection of the same concentration of LPS into the yolk induces high mortality and pathological migration as well as accumulation of neutrophils and macrophages after 48 h. These results demonstrate that LPS injection is a suitable manner for inducing inflammation in the zebrafish model.

### 2.3. Stage for LPS Injection

**Figure 6:**
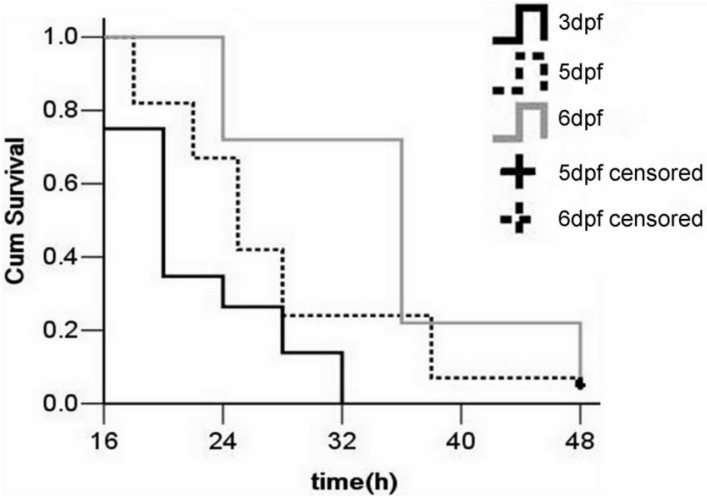
Average cumulative mortality (percent) of zebrafish larvae injected with LPS at different stages. Two-hundred larvae were randomly divided into four groups (*n* = 50 for each). 0.5 mg/mL LPS was injected into the yolk at 3-, 5-, and 6-dpf, and larvae were then monitored for 48 h. Larvae injected with PBS served as negative controls. Each curve represents data pooled from three independent experiments.

Zebrafish neutrophils and arch-macrophages can be detected as early as 18 h post-fertilization (hpf). In the phylotypic-stage (24–48 hpf), the integral blood circulation and a natural immune system in zebrafish have been developed. The components of acquired immunity and primary organs were formed in the hatching stage (48–72 hpf) [23,30,31]. To determine the optimal stage for injection, identical doses of LPS were injected into the yolk at different larval stages (Figure 6). Injections at 3-dpf caused 100% mortality at 32 hpi, while injections at 5- and 6-dpf caused 94% mortality at 48 hpi. All PBS-injected larvae survived (data not shown). The median survival times of larvae injected at 3, 5, and 6-dpf were 20 ± 0.6, 25 ± 0.8, and 36 ± 1.4 h, respectively. The *p*-values for the statistical tests and multiple comparisons were all below 0.001. It suggested that the survival time of the LPS-injected zebrafish larvae increased with the stage at which they were injected. Performing injections at 3-dpf is therefore preferable, as this stage adequately models the immune response but shortens the time required for the experimental analysis.

### 2.4. Effect of LPS Injection Dose on Inflammation

**Figure 7:**
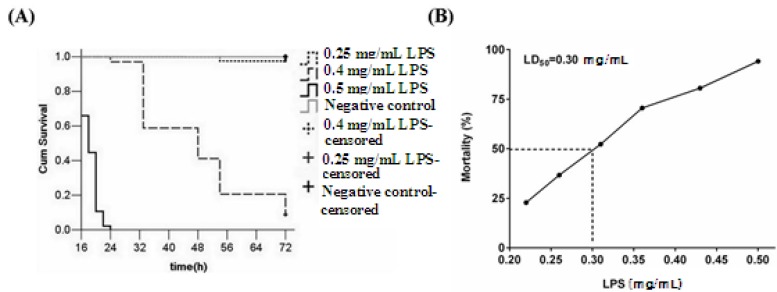
Average cumulative mortality (percent) of zebrafish larvae at different LPS concentrations (A) and the relationship between LPS concentration and mean larval mortality (B). One hundred and forty larvae were randomly divided into four groups (*n* = 35). The yolks of larvae were injected at 3-dpf with the indicated doses of LPS, and larvae were monitored for 48 h. Negative controls were larvae injected with PBS. Each curve represents data pooled from three independent experiments.

The biological activities of LPS are greatly influenced by its source and degree of dissociation in aqueous solution. In order to determine whether the phenotypes observed in larvae depend on the concentration and dose of LPS, a range of doses were injected into the yolk of 3-dpf larvae. The results showed that the injection of 0.5, 0.4, and 0.25 mg/mL LPS caused 100%, 41.2%, and 0% mortality at 48 h, respectively (Figure 7A). With lower LPS concentrations, mortality was decreased while survival time was lengthened. This indicates that 3-dpf larvae are affected by LPS in a dose-dependent manner. 

Based on our preliminary experiments of LPS stimulation, we determined the LD_50_ of LPS at 48 hpi of 3-dpf larvae (Table 3). A Chi-squared test of the dose-effect relationship regression line showed a good fit (χ^2^ = 1.502, *p* = 0.826). The LD_50_ was 0.30 mg/mL (Figure 7B; 95% confidence interval: 0.29, 0.31). LD_99_ was 0.50 mg/mL (95% confidence interval: 0.50, 0.60). As a high mortality rate for the model could greatly reduce the false positives during drug screening, injection doses close to LD_99_ were preferable.

**Table 3:** LD_50_ statistics of LPS.

Group	Grand Total	Death Count
0.500 mg/mL LPS	96	93
0.425 mg/mL LPS	95	86
0.361 mg/mL LPS	96	76
0.307 mg/mL LPS	98	57
0.261 mg/mL LPS	97	29
0.222 mg/mL LPS	99	11
^a^ Negative controls	100	0

^a^ Negative controls were performed using PBS.

### 2.5. Effect of Injection time for the Genes Expressions of Inflammation Cytokines

**Figure 8:**
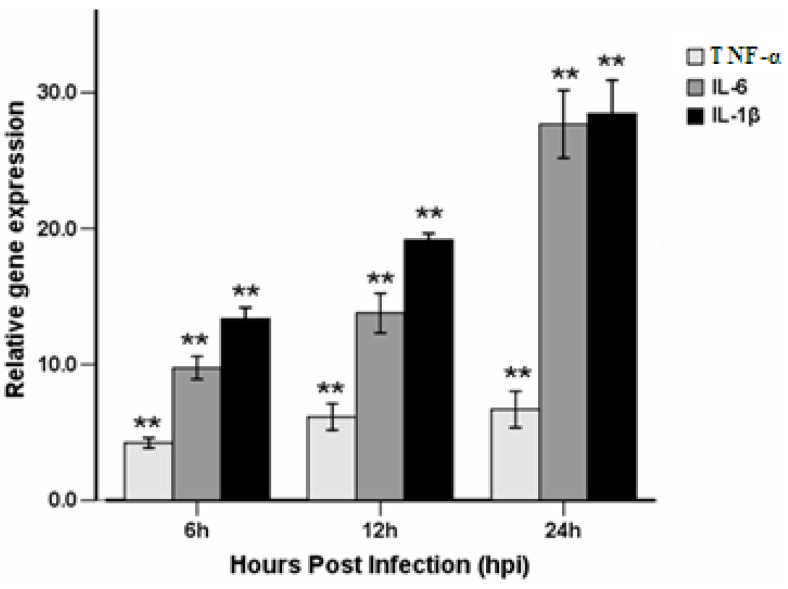
Effect of injection time for the genes expressions of inflammation cytokines by RT-PCR. Expressions of TNF-α, IL-6, and IL-1β gene in 0.5 mg/mL LPS-infected larvae compared with controls determined by quantitative real-time PCR. Larvae were injected with 0.5 mg/mL LPS at 3 dpf. Total RNA was extracted from larvae at 6, 12, and 24 hpi. Each bar represents the mean of three replicates. Error bars represent standard deviation.

The mRNA expression levels of the key cytokines involved in the inflammatory response, including TNF-α, IL-6, and IL-1β, were measured in the control and LPS-injected larvae using quantitative reverse transcription polymerase chain reaction (RT-PCR). The larvae used for quantitative gene expressions were injected at 3-dpf and sampled at 6, 12, and 24 hpi (Figure 8). IL-1β expression levels in LPS-injected larvae showed an immediate response, with a 13-fold increase over control levels at 6 hpi. At 12 and 24 hpi, IL-1β gene expression was increased by 19- and 28-fold, respectively, when compared with the control samples. Expression levels of IL-6 showed similar increases. At 6, 12, and 24 hpi, IL-6 gene expression was 9.7-, 13.9-, and 27.6-fold greater than the controls, respectively. TNF-α expression levels were increased to a lesser extent compared with IL-1β and IL-6 at these time points. At 6 hpi, TNF-α gene expression levels in the LPS-injected larvae were approximately 4.2-fold higher than the controls. At 12 and 24 hpi, TNF-α gene expressions were increased by 6.1- and 6.6-fold, respectively. These data show that the injection of 0.5 mg/mL LPS into the yolk of 3-dpf larvae can be used to induce inflammation responses through up-regulating inflammation cytokines. Of note, our previous studies evaluated the effect of inflammation inducers including species 055:B5 (L2880) or 0111:B4 (L2630, Sigma, St. Louis, MO, USA) on efficacy in zebrafish.

### 2.6. Effectiveness of CA on Reducing LPS-Induced Acute Inflammation in Zebrafish

Chlorogenic acid (CA) is a major component of the dry alabastrum of *Lonicera japonica Thunb.* It has strong anti-inflammatory effects in LPS-induced acute lung injury in mammals, such as mitigating leukocyte infiltration and reducing cytokine expression [32,33]. It also shows a potential anti-edematogenic and anti-nociceptive activities in animal models of carrageenan-induced inflammation and formalin-induced pain, respectively [34]. Therefore, we evaluated the anti-inflammatory effects of CA in our zebrafish model. In this experiment, 3-dpf zebrafish were injected with 0.5 mg/mL LPS (2 nL volume per larva) into the yolk to induce inflammation. At 0.5 hpi, groups were injected with CA (20 mg/mL) or DEX (0.15 mg/mL) (1 nL volume per larva). All groups (PBS, LPS, LPS + DEX, LPS + BCA) were then maintained at 28.5 °C.

#### 2.6.1. Macrophage Recruitment is Reduced by CA Treatment

**Figure 9:**
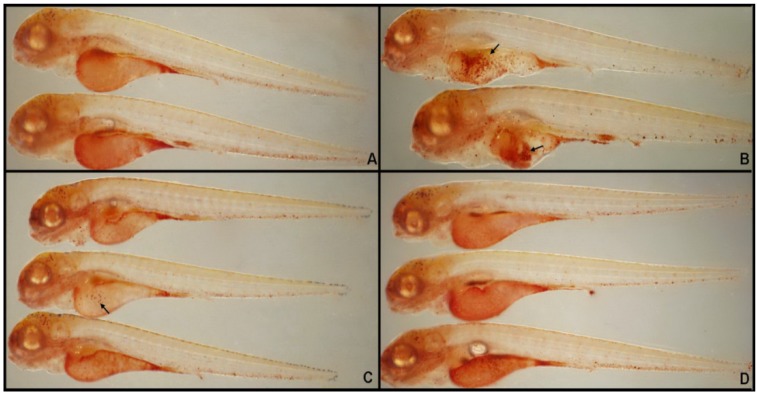
NR labeling of macrophages at 3.5 dpf demonstrates that CA (20 mg/mL) or DEX (0.15 mg/mL, 1 nL volume per larva) significantly reduce LPS-induced macrophage recruitment. (**A**) PBS-injected negative controls; (**B**, arrow) macrophage recruitment to the yolk after LPS-injection; (**C**) DEX-treated, LPS-injected positive controls; and (**D**) CA-treated, LPS-injected larvae.

LPS induces a strong and significant recruitment of macrophages to the yolk (Figure 9B, arrow). NR staining in LPS-injected larvae significantly increased the recruitment of macrophages to the injection site at 14 hpi, compared with PBS-injected controls (Figure 9A). However, the treatment with CA (20 mg/mL) had significantly reduced macrophage recruitment (Figure 9D). Furthermore, treatment with a positive control, DEX (0.15 mg/mL), caused a similar reduction in macrophage recruitment (Figure 9C). These results indicated that CA held a significant anti-inflammatory activity in our zebrafish model.

#### 2.6.2. The Reduction of CA Treatment on Neutrophil Recruitment

**Figure 10:**
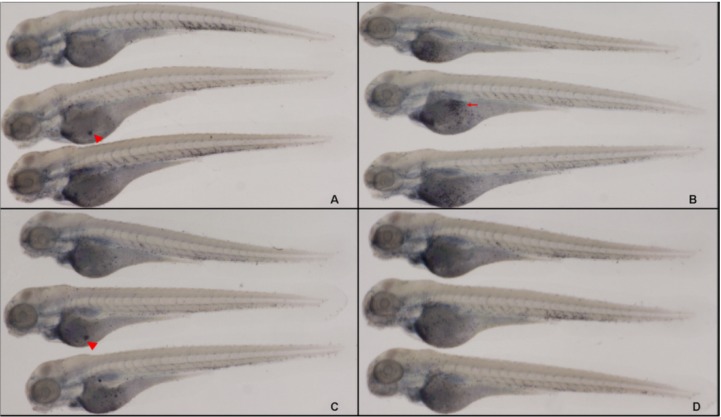
SB labeling of neutrophils at 3 dpf demonstrates the anti-inflammatory effect of CA: both CA (20 mg/mL) and DEX (0.15 mg/mL) significantly reduce the neutrophil recruitment induced by LPS. (**A**) PBS-injected negative controls (arrowhead indicates non-specific signal); (**B**, arrow) neutrophil recruitment significantly increased at 6 hpi after LPS-injection; (**C**) DEX-treated, LPS-injected positive controls; and (**D**) CA-treated, LPS-injected larvae.

**Figure 11:**
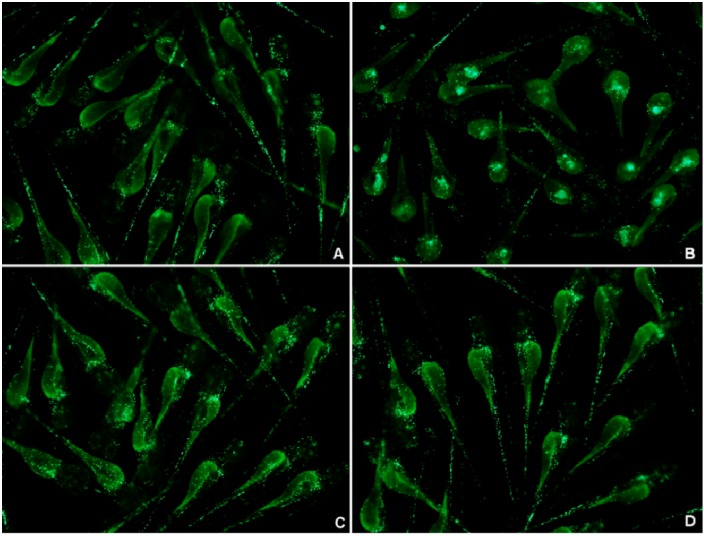
MPO:GFP transgenic line allows tracking of neutrophils at 3.5 dpf and demonstrates the anti-inflammatory effect of CA. CA (20 mg/mL) and DEX (0.15 mg/mL) significantly reduce neutrophil recruitment and attenuate tissue necrosis induced by LPS. (**A**) PBS-injected negative controls; (**B**) neutrophil recruitment to the yolk and necrosis in LPS-injected controls; (**C**) DEX-treated, LPS-injected positive controls; and (**D**) CA-treated, LPS-injected larvae.

LPS induced an acute inflammatory response which was characterized by the recruitment of neutrophils to the yolk (Figure 10B, arrow). SB staining showed a significant increase in LPS-induced neutrophil recruitment at 6 hpi compared with the PBS-injected controls (Figure 10A, arrowhead is non-specific staining). Both CA (Figure 10D) and DEX treatment (Figure 10C, arrow is the neutrophil, arrowhead is non-specific staining) significantly reduced neutrophil migration to the LPS injection site. The MPO:GFP transgenic line, which allowed the live tracking of neutrophils, showed similar results (Figure 11). Each larva in the untreated LPS-injected group (sham group) showed a massive recruitment of neutrophils and yolk necrosis, while this was barely noticeable in either CA- or DEX-treated larvae (Figure 10D and Figure 11C). These results demonstrate that CA treatment can inhibit neutrophil recruitment and yolk necrosis during LPS-induced acute inflammation in our model.

#### 2.6.3. Survival Analysis of CA-Treated Larvae

**Figure 12:**
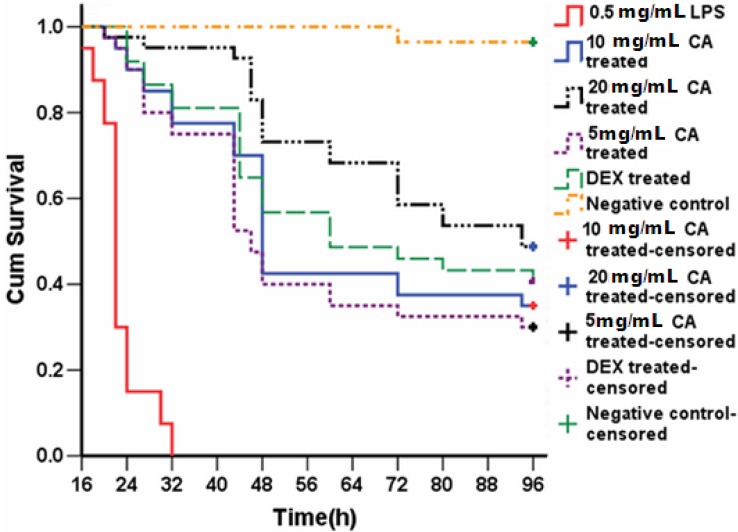
Protective effect of CA (20 mg/mL) on 3-dpf zebrafish larvae challenged with LPS. Three-hundred larvae were randomly divided into six groups (*n* = 50). The yolks of larvae were injected with 0.5 mg/mL LPS at 3-dpf and treated with the indicated doses of CA. Mortality was monitored for 96 h. Negative controls were injected with PBS. Each curve represents data pooled from three independent experiments.

To test whether the profound anti-inflammatory effects of CA are concentration dependent, a range of doses were injected into the yolk at 0.5 h after injection of 0.5 mg/mL LPS into 3-dpf larvae. Larvae were then monitored for signs of disease and mortality for 96 h (Figure 12). LPS injection alone caused 100% mortality within 32 h. In contrast, treatment of LPS-injected larvae with DEX or CA attenuated mortality by 25%. A dose-dependent increase in survival was seen with increasing doses of CA, with up to 95% survival at 32 h using 20 mg/mL CA (Figure 12). After 48 h these larvae showed a survival rate generally greater than 75%; and 48.8% after 96 h. The survival curves indicated that all embryos were alive until 20-h post-treatment in all of the CA treatment groups. After this point, the embryos fell into one of two groups; they were either dead (associated with severe malformation) or alive until the end of the study. The median survival times of the larvae treated with 5, 10, and 20 mg/mL of CA were 46, 48, and 94 h, respectively; much longer than the 22 h of the untreated LPS-injected larvae. 

### 2.7. Discussion

Studies have shown that the immune system of zebrafish was remarkably similar to that of humans [35,36,37], displaying similarities in inflammatory responses, including vascular cell migratory/phagocytic processes, and inflammatory factors. Pathological immunocyte migration is an important sign of an inflammatory response. Therefore, the migration of phagocytes can be visualized using a simple staining protocol in zebrafish or transgenic lines. These monitoring systems are both convenient and inexpensive. Using mortality as a primary end-point is also extremely useful and easily scored. This approach is rapid, cheap, and efficient.

The key variables including the LPS species and concentration were associated closely with the model in LPS-induced acute inflammation. Zebrafish have different sensitivities towards different LPS species. Additionally, LPS is a polymer in aqueous solution and its degree of dissociation significantly affects its biological activity. In mammals, candidate drugs have been used in combination with the LD_50_ of toxic agents in the screening process, which allows for more thorough investigation of promising candidates using fewer animals. Such an experimental set-up can reduce costs, but increase the observation time and screening cycle. Furthermore, it increases the likelihood of false positives. 

The mass migration of macrophages and neutrophils occurs rapidly towards the site of LPS injection, accompanied by the up-regulation of IL-1β, IL-6, and TNF-α expression. These results provide evidence that the inflammatory immune response is stimulated by LPS microinjection. These cytokines play a critical role in initiating the proinflammatory cytokine cascade, activating and recruiting macrophages and neutrophils, and stimulating the adaptive immune response. The injection of LPS into the yolk also caused characteristic signs of endotoxic inflammation, including phagocyte migration, yolk necrosis, and hemorrhagic pericardium at 24 h. These results suggest that zebrafish are suitable for the model of LPS-triggered infection. Further, the zebrafish larval yolk might be a site of relatively high immune reactivity, with a physical structure (*i.e.*, plasma membrane) that functions to sense LPS and trigger the chemotaxis of macrophages and neutrophils to limit the impact of infection [38].

NR staining can efficiently label macrophages in live zebrafish at 3 dpf [25]. This protocol allows the observation of macrophage chemotaxis to the site of injection, and is rapid and convenient. However, a limitation of this technique is that macrophages can only be observed over a limited period of time, which does not permit their tracking throughout the whole process. SB staining has the same limitation. The MPO:GFP transgenic line, in contrast, allows the visualization of neutrophil chemotaxis during the entire process of inflammation. In order to validate this screening method, we used both SB staining and the MPO:GFP line to detect the recruitment of neutrophils after LPS injection. The two approaches yielded similar results. In combination with NR staining, these methods allowed the comprehensive study of leukocyte migration *in vivo* during LPS-induced inflammation in our zebrafish model.

It has been reported that the retrograde chemotaxis of neutrophils may complement the regulated apoptosis of neutrophils as a mechanism to resolve inflammation [39]. The MPO:GFP line revealed that neutrophils at the wound site in PBS-injected larvae were disappeared by 6 hpi. In contrast, a great number of neutrophils were recruited to LPS-injection foci at 6 hpi, where fluorescent signals remained until 12 hpi. Taken together, these observations suggest that zebrafish larvae recover from the trauma caused by a needle at around 6 hpi. However, “round-trip” migration did not occur after LPS stimulation, where large neutrophils remained at the wound site [39]. The residual fluorescent signal foci might represent necrotic tissue forming as a result of neutrophil necrosis, which released phagosomes and hydrolytic enzymes to dissolve the surrounding tissue.

The survival test is one of the most commonly used methods to screen for the activity of acute anti-inflammatory drugs in animals. The survival rate and median survival time after treatment are useful primary end-points to evaluate drug activity. The TCM anti-inflammatory agent CA, at doses of 5 and 20 mg/mL, effectively inhibited phagocyte recruitment, improved survival, and lengthened median survival time. Our results showed that CA had a similar effect as DEX. These data indicate that CA and DEX exhibit anti-inflammatory activities during endotoxic inflammation in our zebrafish model. Nevertheless, the zebrafish model also has some disadvantages. Factors such as egg quality affect the experimental system. For example, female fish that do not breed for long periods of time tend to lay dead eggs and yield poor quality larvae. This requires the selection of appropriate larvae from a female fish spawning over a minimum of 2–3 weeks. Further, non-automated microinjection limits the implementation of high-throughput drug screens. Finally, in order to obtain a high mortality rate, we chose a high dose of LPS (LD_99_) in this study in order to greatly reduce false positives. This protocol, however, may fail to identify weakly effective drugs, resulting in false negative results.

## 3. Experimental

### 3.1. Chemicals and Reagents

Chlorogenic acid (CA, Batch No.110753-200413, purity > 98%) and dexamethasone (DEX) were purchased from the National Institute for the Control of Pharmaceutical and Biological Products (Beijing, China). Methylene blue, 1-phenyl-2-thiourea (PTU), neutral red, LPS (E. coli, 055: B5, L2880; 0111:B4, L2630; total impurities < 3% protein) were ordered from Sigma Chemical (St. Louis, MO, USA). SB was obtained from Acros Organics (Morris Plains, NJ, USA) while RNAiso Plus from Takara Bio INC. (Otsu, Shiga, Japan). RNAlater RNA stabilization solution was from Ambion Inc., (Austin, Texas, TX, USA). Remaining reagents were AR grade and commercial sources.

### 3.2. Zebrafish Embryo and Larvae Maintenance

The transgenic zebrafish MPO:GFP line [17] was obtained from the Key Laboratory of Zebrafish Modeling and Drug Screening for Human Diseases Institute, Southern Medical University (Guangzhou, China). Zebrafish embryos were maintained and raised according to the protocol described by Westerfield [40]. Zebrafish were kept at 28.5 °C with a 14:10-h light:dark cycle in a recirculating tank system using local tap water (pH 7.2–7.6, salinity 0.03%–0.04%). Fertilized embryos collected after natural spawning were cultured at 28.5 °C in clean Petri dishes in egg water containing 60 mg/L “Instant Ocean” salt and 2 mg/L methylene blue (Sigma). To inhibit melanin formation, 0.003% 1-phenyl-2-thiourea (PTU) (Sigma) was added to the egg water after 10–12 h. The MPO:GFP line expresses green fluorescent protein in neutrophils under the control of the myeloperoxidase promoter (MPO), which allows *in vivo* tracking of neutrophil migration during the acute inflammatory reaction.

### 3.3. Routes of Delivery: Exposure by Immersion or Microinjection

To eliminate differences arising from varying parentage, all zebrafish larvae were divided randomly into experimental and control groups. For exposure by immersion, larvae were treated with 0.5 mg/mL LPS by static immersion for 48 h in a total volume of 3 mL. Control larvae were exposed to regular egg water.

For exposure by microinjection, larvae were anesthetized using 0.02% tricaine. LPS (0.5 mg/mL) was injected into the yolk. A negative control group was injected with phosphate-buffered saline (PBS). Microinjection (Narshige PLI-100, Harvard Apparatus, Inc., Massachusetts, MA, USA) was performed with a volume of 2 nL per larva. Dead larvae were removed within 0.5 hpi. Treatments were performed in quadruplicate (34 larvae per group per dose). Each group of larvae was then cultured at 28.5 °C and observed for signs of disease and mortality. Larvae from each replicate group were sampled by staining with NR and SB, and for survival.

### 3.4. Neutral Red Staining

Neutral red is a vital dye that accumulates in the lysosomes through endocytosis. As macrophage cells undergo efficient endocytosis, NR labels macrophages more robustly than any other cell types. Optimal staining of macrophages in live embryos was achieved by incubating embryos in 2.5 μg/mL NR solution containing 0.003% PTU at 28.5 °C in the dark for 6–8 h [25]. After staining, macrophage migration was observed using a dissecting microscope (IX71, Olympus, Tokyo, Japan).

### 3.5. Sudan Black Staining

Sudan black (SB) is an azo stain that detects the presence of lipids, with dark staining representing the cytolymph. Staining also occurs in neutrophils more than other cell types. A stock solution of SB was prepared from SB powder (0.6 g) dissolved in pure ethanol (200 mL), filtered, and stored at 4 °C. A buffer solution was made from phenol (16 g) dissolved in pure ethanol (30 mL) plus Na_2_HPO_4_**·**12H_2_O (0.3 g) dissolved in distilled water (100 mL). A working staining solution was made by mixing stock solution (30 mL) with buffer (20 mL), and filtering. Whole larvae were fixed with 4% methanol-free paraformaldehyde (PFA) in PBS for 2 h at room temperature, rinsed in PBS, incubated in SB for 40 min, washed extensively in 70% ethanol in water, then progressively rehydrated with PBS plus 0.1% Tween-20 (PBT) [41].

### 3.6. Timing of LPS Injection

Two hundred zebrafish larvae from the same mother were randomly divided into four groups: PBS-injected negative controls, and larvae injected with LPS at 3, 5, and 6-dpf (50 larvae per group). 0.5 mg/mL LPS (2 nL) was injected into the yolk of each larva. Larvae were then cultured at 28.5 °C and observed for signs of disease and mortality for 48 h.

### 3.7. LPS Injection Doses

In preliminary experiments, zebrafish larvae were injected with 0.25, 0.4, or 0.5 mg/mL LPS. The concentration of LPS that elicited 50% mortality (LD_50_) was calculated by nonlinear regression:

Y/Y_max_ = {1 − [D / (D + LD_50_)]n} (1)

where Y is the response to LPS-induced mortality, Y_max_ is the maximal response without LPS, D is the concentration of LPS, and n is the Hill coefficient. Larvae were divided randomly into six groups and injected with 0.222, 0.261, 0.307, 0.361, 0.425, or 0.5 mg/mL LPS. A negative control group was injected with an equal volume of PBS. Microinjection volume was 2 nL per larvae. All larvae were cultured at 28.5 °C and observed for signs of disease and mortality for 48 h. Statistics were calculated and graphs were constructed using GraphPad Prism 5.0.

### 3.8. Quantitative Real-Time PCR

Total RNA was extracted from zebrafish larvae at 3-dpf which were injected with PBS or LPS (0.5 mg/mL) at 6, 12, and 24 hpi. Larvae were stored in RNAlater RNA stabilization solution at 4 °C until samples from all the time points were collected.

PCR primers used in this study were designed from their respective gene sequences using the Primer 3 program. Primer sequences were purchased from Invitrogen, Forward and reverse Primer sequences (5'-3') were: β-actin, ATGGATGAGGAAATCGCTG, ATGCCAACCATCACTCCCTG; TNF-α, GCTG GATCTTCAAAGTCGGGTGTA, TGTGAGTCTCAGCACACTTCCATC; IL-1β, TGGACTTCGCAGCACAAAATG, GTTCACTTCACGCTCTTGGATG; IL-6, AGACCGCTGCCTGTCTAAAA, TTTGATGTCGTTCACCAGGA. Total RNA was extracted using RNAiso Plus (Takara, Japan) and reverse-transcribed into cDNA. Each reverse transcription reaction contained total RNA (1 µg), 5 × PrimeScript Buffer (2 µL), PrimeScript RT Enzyme mix (0.5 µL), oligo dT primer (25 pM), and random 6-mers (50 pM). Samples were incubated at 42 °C for 30 min, heat-inactivated at 85 °C for 5 s, and then diluted in 10 volumes of water.

Target cDNA was amplified using the SYBR green PCR core reagent kit (Takara). Each reaction contained SYBR Premix Ex Taq^TM^ (10 µL), forward and reverse primers (160 nM), and cDNA (0.6 µL). Thermocycling conditions were an initial denaturation at 95 °C for 10 min, 95 °C for 10 s, and 60 °C for 30 s. All reactions were performed in triplicate for 40 cycles in a Stratagene Mx3005P system. Relative quantitation was performed using melt curves for quality control. The relative quantity of gene expression in each sample was analyzed by the 2(-ΔΔCt) method with normalization to the level of β-actin.

### 3.9. Statistical Analysis

Survival experiments were evaluated using the Kaplan–Meier method. Comparisons between the curves were made using the Log-rank test. The data of occurrence rate are expressed as mean ± standard error of the mean (SEM). The LD_50_ experiment was evaluated using the Bliss method. Statistical analysis was performed using SPSS13.0. *p*-value less than 0.05 was considered as a statistical significant.

## 4. Conclusions

Overall, this work confirms that zebrafish could be utilized for the rapid screening of drugs against endotoxin-induced inflammation. Our results also showed that CA displays potential anti-inflammatory activity. All results indicated that this model may enable the evaluation and screening of active components of TCM and suitable for commercial development.
